# Tip-detection antegrade dissection and re-entry into a seriously collapsed true lumen

**DOI:** 10.1093/ehjcr/ytae373

**Published:** 2024-08-14

**Authors:** Yutaka Tadano, Takuro Sugie, Daitaro Kanno, Tsutomu Fujita

**Affiliations:** Department of Cardiovascular Medicine, Sapporo Cardio Vascular Clinic, Sapporo Heart Center, North 49, East 16, 8-1, Higashi Ward, 007-0849, Sapporo, Japan; Department of Cardiovascular Medicine, Sapporo Cardio Vascular Clinic, Sapporo Heart Center, North 49, East 16, 8-1, Higashi Ward, 007-0849, Sapporo, Japan; Department of Cardiovascular Medicine, Sapporo Cardio Vascular Clinic, Sapporo Heart Center, North 49, East 16, 8-1, Higashi Ward, 007-0849, Sapporo, Japan; Department of Cardiovascular Medicine, Sapporo Cardio Vascular Clinic, Sapporo Heart Center, North 49, East 16, 8-1, Higashi Ward, 007-0849, Sapporo, Japan

An 80-year-old man with effort angina underwent an elective intervention for right coronary artery stenosis. A large right ventricle branch was occluded after stenting (*Figure 1A* and *B*). Two hours later, the patient developed ventricular fibrillation. After direct current, systolic blood pressure remained low under vasopressor administration with the suspected diagnosis of isolated right ventricular infarction, prompting us to perform an urgent intervention (see Supplementary material online, *Video S1*). After a guidewire was advanced in a false lumen of the right ventricular branch, the bailout sub-intimal tracking and re-entry technique was unsuccessful (*Figure 1C*; Supplementary material online, *Video S2*). The true lumen collapsed until it became crescent shaped on intravascular ultrasound (IVUS) images (*Figure 1D*; Supplementary material online, *Video S3*). Having no visible distal target and collaterals, we attempted tip-detection antegrade dissection and re-entry (TDADR) as a bailout (*Figure 1E*) and punctured the true lumen using the dedicated guidewire Conquest Pro 12 ST (ASAHI INTECC, Japan), which was intentionally advanced through to extra-vessel (*Figure 1F–H*). We advanced the micro-catheter within the true lumen based on the IVUS finding (i.e. ‘blackening’ around the guidewire). A swapped polymer-jacketed guidewire passed through successfully. After IVUS confirmation (*Figure 1I–K*; Supplementary material online, *Video S4*), a 2.25/38 mm stent was placed. Intra-aortic balloon pumping was inserted due to slow flow (*Figure 1L*; Supplementary material online, *Video S5*). The patient was discharged on dual antiplatelet therapy 3 months later.

**Figure 1:**
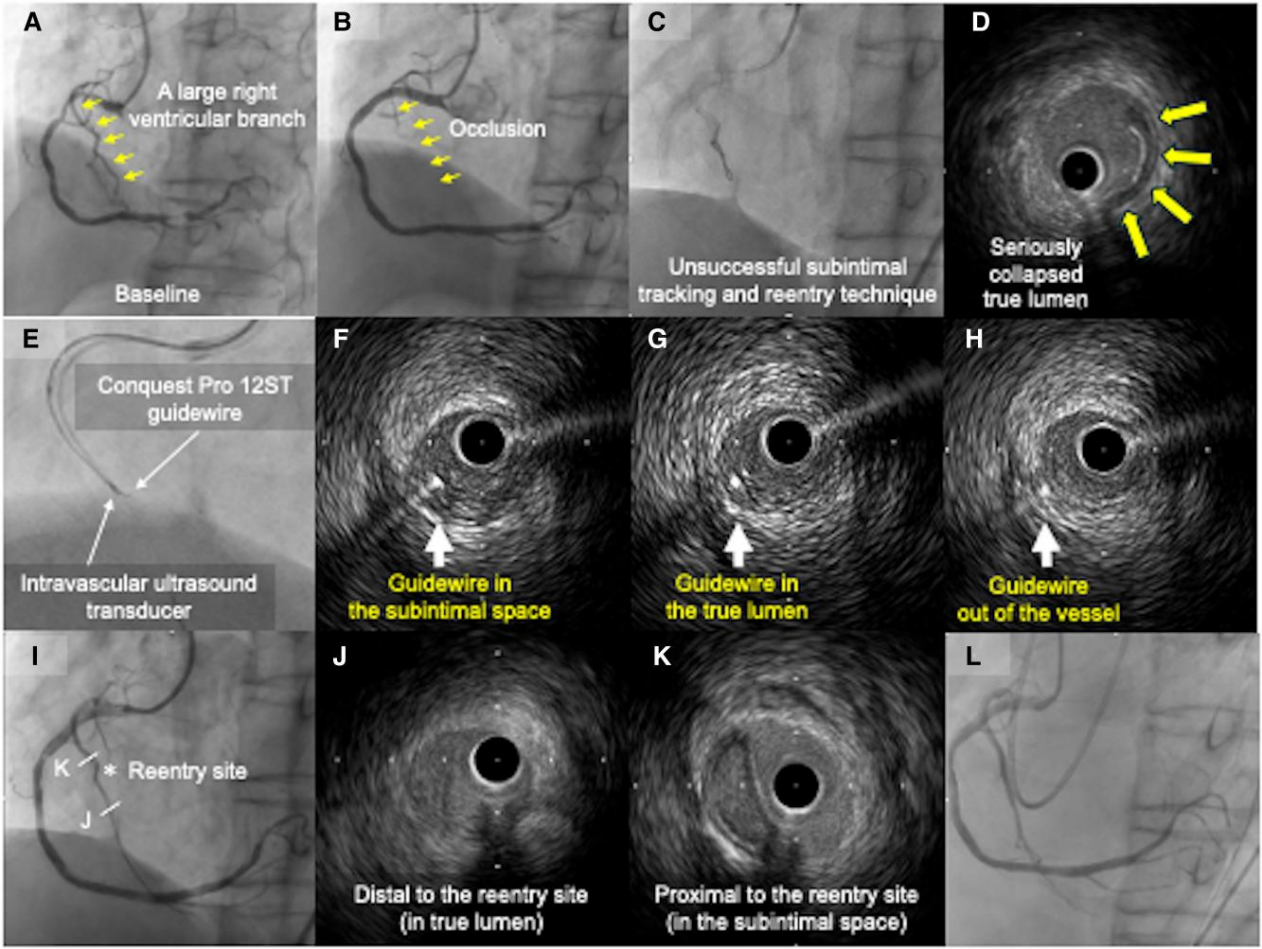
Images during the tip-detection antegrade dissection and re-entry into a seriously collapsed true lumen. (*A*) Baseline angiography. (*B*) A large right ventricular branch had occluded (small arrows). (*C*) Bailout sub-intimal tracking and re-entry technique was unsuccessful. (*D*) The true lumen became seriously collapsed. (*E–H*) Images during the TDADR. (*I*) The asterisk is the re-entry site of the TDADR. (*J*) The intravascular ultrasound image, distal to the re-entry site, shows the true lumen route and (*K*) the sub-intimal route proximal to it. (*L*) The angiography on the following day.

Tip-detection antegrade dissection and re-entry can be applied to occlusions with an angiographically invisible distal target unlike device-based ADR, although both the dedicated guidewire and a second operator with excellence in IVUS are needed.^1,2^ In addition, unlike the retrograde approach, TDADR can be performed under no collaterals available. The weakness of TDADR is that calcified sites should be avoided; however, the risk of coronary perforation is not higher compared with conventional antegrade wiring.

## Supplementary Material

ytae373_Supplementary_Data

## Data Availability

The data underlying this article are available in the article and in its online Supplementary material.

## Notes

**Consent:** The authors confirm that written consent for submission and publication of this case report has been obtained from the patient in line with COPE guidance.

**Funding:** None declared.
